# Ayurveda and cancer

**DOI:** 10.4103/0974-8490.75463

**Published:** 2010

**Authors:** Roopesh Jain, Susmit Kosta, Archana Tiwari

**Affiliations:** *School of Biotechnology, Rajiv Gandhi Proudyogiki Vishwavidyalaya, Gandhi Nagar, Bhopal, Madhya Pradesh, India*

Sir,

Cancer, one of the most deadly challenges spreading drastically in 21^st^ century, has now officially become the most dangerous killer in the world according to the World Health Organization. Who can deny the fact that cancer is related to adversary of modernization and advanced pattern of irregular and stressed life dominated by Western medicine. Scientists are making their best efforts to fight this disease; however the sure-shot cure is still awaited.

Ayurveda, the oldest Indian indigenous medicine system of plant drugs is known from very early times for preventing or suppressing various tumors using these natural drugs. And nowadays scientists are keener to researches on complementary and alternative medicine for the management of cancer. In Ayurvedic concept, according to ‘Charaka’ and ‘Sushruta Samhitas’ cancer is described as inflammatory or non-inflammatory swelling and mentioned either as ‘Granthi’ (minor neoplasm) or ‘Arbuda‘ (major neoplasm).[1] The nervous system (Vata or air), the venous system (Pitta or fire) and the arterial system (Kapha or water) are three basics of Ayurveda and very important for normal body function. In malignant tumors all three systems get out of control (Tridoshas) and lose mutual coordination that causes tissue damage, resulting critical condition. Tridoshas cause excessive metabolic crisis resulting in proliferation.[2]

The modern cancer therapy which is known to burdened by drug-induced toxic side effects hoping perfect cure of disease form the complementary and alternative medicine system. The main goal of Ayurvedic therapy is to find the ultimate cause of an illness while the therapeutic approach of Ayurveda is divided into four categories as Prakritisthapani chikitsa (health maintenance), Rasayana chikitsa, (restoration of normal function), Roganashani chikitsa (disease cure) and Naishthiki chikitsa (spiritual approach).[3] Commonly used herbal decoctions reported in Ayurveda are made of multiple herbs possessing great potential for a cancer cure; scientifically these formulations work on multiple biochemical pathways and influence different organ systems all together and nourish the body as a whole by supporting body‘s deference systems.

Herbs help total healing, reduces the side effects and cancer-associated complications.[4] *Andrographis paniculata, Annona atemoya, Phyllanthus niruri, Piper longum, Podophyllum hexandrum, Tinospora cordifolia, Semecarpus anacardium, Vitis vinifera, Baliospermum montanum, Madhuca indica, Pandanus odoratissimum, Pterospermum acerifolium, Raphanus sativus, Barleria prionitis, Prosopis cineraria, Amorphopallus campanulatus, Oxoxylum indicum, Basella rubra, Flacourtia romantchi, Moringa oleifera, Ficus bengalensis, Curcuma domestica, Allium sativum, Calotropis gigantean, Datura metel, Hygrophila spinosa, Juniperus indica, Moringa oleifera, Nigella sativa, Picrorrhiza kurroa, Rubia cordifolia,* etc. are various plants having scientific evidence of anticancer property. Nowadays, many herbs are under clinical studies and being investigated phytochemically to understand their anticancer potential. More than 25% of drugs used during the last 20 years are directly derived from plants, while the other 25% are chemically altered natural products. Nine plant-derived compounds including vinblastine, vincristine, etoposide, teniposide, taxol, navelbine, taxotere, topotecan and irinotecan have been approved for use as anticancer drugs. 10-hydroxycamptothecin, monocrotaline, d-tetrandrine, lycobetaine, indirubin, colchicinamide, curcumol, curdione, gossypol and homoharringtonine are few more plant-derived compounds of high hope.[5,6]

Each herb contains multiple active principles that often operate synergistically producing therapeutic benefits and lowering the risks of adverse effects; and avoids the need for supplemental therapy to manage cancer cachexia. Now it is important to raise awareness and encourage implementation of Ayurvedic therapies for combating cancer and suggest an integrated approach in tumor management and treatment.
